# Management and Outcome of Women with Placenta Accreta Spectrum and Treatment with Uterine Artery Embolization

**DOI:** 10.3390/jcm13041062

**Published:** 2024-02-13

**Authors:** Vanessa Neef, Armin N. Flinspach, Katrin Eichler, Tirza R. Woebbecke, Stephanie Noone, Jan A. Kloka, Lukas Jennewein, Frank Louwen, Kai Zacharowski, Florian J. Raimann

**Affiliations:** 1 Department of Anaesthesiology, Intensive Care Medicine and Pain Therapy, University Hospital, Goethe University Frankfurt, Theodor-Stern-Kai 7, 60590 Frankfurt, Germany; flinspach@med.uni-frankfurt.de (A.N.F.); woebbecke@med.uni-frankfurt.de (T.R.W.); stephanie.noone@med.uni-frankfurt.de (S.N.); jan@dsgfrankfurt.de (J.A.K.); zacharowski@med.uni-frankfurt.de (K.Z.); raimann@med.uni-frankfurt.de (F.J.R.); 2 Department of Interventional Radiology, University Hospital, Goethe University Frankfurt, Theodor-Stern-Kai 7, 60590 Frankfurt, Germany; k.eichler@em.uni-frankfurt.de; 3 Department of Obstetrics and Perinatal Medicine, University Hospital, Goethe University Frankfurt, Theodor-Stern-Kai 7, 60590 Frankfurt, Germany; jennewein@med.uni-frankfurt.de (L.J.); louwen@em.uni-frankfurt.de (F.L.)

**Keywords:** placenta accreta spectrum, postpartum hemorrhage, blood transfusion, pregnancy, uterine artery embolization

## Abstract

Background: Placenta accreta spectrum (PAS) disorders are a continuum of placental pathologies with increased risk for hemorrhage, blood transfusion and maternal morbidity. Uterine artery embolization (UAE) is a safe approach to the standardization of complex PAS cases. The aim of this study is to analyze anemia and transfusion rate, outcome and anesthesiological management of women who underwent caesarean delivery with subsequent UAE for the management of PAS. Material and Methods: This retrospective observational study included all pregnant women admitted to the University Hospital Frankfurt between January 2012 and September 2023, with a diagnosis of PAS who underwent a two-step surgical approach for delivery and placenta removal. Primary procedure included cesarean delivery with subsequent UAE, secondary procedure included placenta removal after a minim of five weeks via curettage or HE. Maternal characteristics, anesthesiological management, complications, anemia rate, blood loss and administration of blood products were analyzed. Results: In total, 17 women with PAS were included in this study. Of these, 5.9% had placenta increta and 94.1% had placenta percreta. Median blood loss was 300 (200–600) mL during primary procedure and 3600 (450–5500) mL during secondary procedure. In total, 11.8% and 62.5% of women received red blood cell transfusion during the primary and secondary procedures, respectively. After primary procedure, postpartum anemia rate was 76.5%. The HE rate was 64.7%. Regional anesthesia was used in 88.2% during primary procedure. Conclusion: The embolization of the uterine artery for women diagnosed with PAS is safe. Anemia management and the implementation of blood conservation strategies are crucial in women undergoing UAE for the management of PAS.

## 1. Introduction

The placenta accreta spectrum (PAS) is defined as a morbidly adherent placenta and includes placenta increta, accreta, and percreta [1]. In recent years, the prevalence of PAS has increased and is currently estimated to be 1 out of 500 births [2]. Despite advances in prenatal ultrasound and diagnosis and referral to experienced surgical centers [3,4], abnormal placentation remains one of the main causes for postpartum hemorrhage (PPH), increased maternal morbidity and mortality [5,6]. Median blood loss during delivery in women diagnosed with PAS is estimated to be 3000 mL [7]. In addition, >90.0% of all women with PAS receive red blood cell (RBC) transfusion during childbirth [8,9]. Planned caesarean hysterectomy (HE) at the time of the delivery is the standard of care [10]. Uterine artery embolization (UAE) is an alternative to caesarean HE, allowing preservation of the uterus and improvement of PPH, recommended by several national and international guidelines [11,12,13].

Previous studies have shown that a multidisciplinary approach in the management of PAS improves maternal outcome [4,14,15,16,17]. A study by Melber et al. on 15 women diagnosed with PAS reveals, that vessel embolization reduces RBC requirements (0.0 (0.0–2.0) vs. 2.0 (0.0–4.5) units; *p* = 0.045) and blood loss (750 (450–1050) vs. 1750 (1050–2500) mL; *p* = 0.003) compared to standard care [18]. Another study by Sebastian et al. demonstrates, that UAE after caesarean delivery can reduce blood loss to 1900 mL and decrease HE rate to 44.0% [19]. These findings are also supported by a recent meta-analysis including 421 women with PAS undergoing UAE. Women with UAE had significantly reduced intraoperative blood loss (*p* = 0.020) and emergency HE (*p* = 0.020) compared to women without UAE [20].

In most cases of caesarean HE, regional anesthesia is performed with conversion to general anesthesia after delivery. Epidurals are often used for postoperative pain management [18]. 

At the University Hospital Frankfurt women with PAS are treated with a specialized and standardized two-step approach: Caesarean delivery with subsequent UAE (primary procedure) and placenta removal after a minimum of five weeks via curettage or HE (secondary procedure). Data on anemia rate and transfusion requirements especially during UAE (primary procedure) and curettage/HE (secondary procedure) are scarce. With the intention to improve the outcome and management of UAE in women with PAS, the present study aims to examine anesthesiological management, anemia and transfusion rates over a period of 12 years.

## 2. Materials and Methods

This retrospective, observational study was conducted at the University Hospital Frankfurt in cooperation with Department of Obstetrics and Perinatal Medicine and the Department of Interventional Radiology. This study was performed according to the principles of the Declaration of Helsinki [21]. The study protocol was approved by the ethics committee of the University Hospital Frankfurt (ref: 2023-1507) and the requirement for written informed consent by the patient was waived.

### 2.1. Patient Data

Data on hospitalized pregnant women (age ≥18 years) diagnosed with PAS and scheduled for caesarean section with subsequent UAE between 1 January 2012 and 30 September 2023 were collected. Data were extracted from the electronic hospital information system Orbis (Agfa HealthCare, Mortsel, Belgium) and GeDoWin (Saatmann GmbH, Worms, Germany). All patients received standard perioperative care. The patient-specific observation period ranged from hospital admission for the primary procedure (including caesarean section and subsequent UAE) until hospital discharge after the secondary procedure (including further vessel embolization (if necessary) and placenta removal via curettage or HE).

Extracted data were preoperative patient specifics like age, body mass index (BMI), the American Society of Anaesthesiologist’s (ASA) physical status score, comorbidities (e.g., pre-eclampsia), hemoglobin (Hb) value at hospital admission for the primary and secondary procedures, number of gravity and parity, previous caesarean sections as well as the type of pregnancy (singleton/twin). PAS-related data included the invasion of neighboring organs and the existence of concomitant placenta previa.

Surgery-related data were hospital length of stay (LOS) for the primary and secondary procedures, intensive care unit (ICU) LOS, blood loss, transfusion of blood products, duration and number of conducted embolizations as well as the name of the embolized vessels.

Anesthesiological variables included the use of venous and arterial catheters (central venous catheter or arterial line) and anesthesiological management (general- or regional anesthesia). Outcome events included HE, pulmonary embolism, stroke, cardiac complication, renal failure, pneumonia, resuscitation, as well as Hb values at hospital discharge after the primary and secondary procedures.

### 2.2. Management of Women Diagnosed with PAS

For primary procedure (caesarean delivery and subsequent UAE), epidural anesthesia is performed; in case of contraindication (e.g., thrombocytopenia), general anesthesia is necessary.

For primary procedure, the patient is monitored using an electrocardiogram (ECG), oxygen saturation and non-invasive or invasive blood pressure measurement. Caesarean section is performed using the modified Misgav Ladach technique. In some cases, a midline skin incision was chosen because the uterotomy had to be performed at the upper uterine body due to placenta localization. Delivery of the baby is conducted with ultrasound-guided incision of the uterus to avoid placental damage. In case of PPH, tranexamic acid (1000 mg) is administered.

After caesarean delivery, the patient is transferred to the radiology department for UAE with presence of the anesthesiologist. Monitoring is conducted with ECG, oxygen saturation and blood pressure.

After embolization, patients were monitored closely for 48 h on the regular ward. Measurements included hourly blood pressure and heart rate as well as continuous measurement of oxygen saturation. Patients are discharged five days after the primary procedure at the earliest. They are followed up by outpatient appointments every three days. Within these appointments, subjective well-being, signs of infection (temperature, leukocyte count), vaginal bleeding and pain are evaluated. Additionally, ultrasound examination is performed at least weakly in order to observe morphogenic changes and vascularization of the placenta. After a minimum of five weeks, patients are scheduled for placenta removal.

For secondary procedure (placenta removal via curettage or HE), either general anesthesia or regional anesthesia is performed. In case of PPH, the patient receives tranexamic acid (1000 mg). In addition, for placenta removal, oxytocin (3–10 IE) is administered, and if necessary, sulprostone (max 1500 μg/24 h).

Depending on the patient’s physical status after the secondary procedure, further monitoring on the ICU may be necessary but not mandatory. After placenta removal, regular postoperative surveillance is conducted. For the patient’s well-being and, if a HE is necessary, with unsuspicious wound healing, patients are dismissed two days after the secondary procedure.

### 2.3. Embolization

Initial femoral access was obtained and an angiography of the aortic bifurcation and the pelvis was conducted using a 5-F pigtail catheter (CORDIS, Miami, FL, USA). The large postpartum uterus showed marked hypervascularity extending into the peri-uterine tissues. Uterine arteries were elongated and dilated. Then, a five-French Cobra catheter (TERUMO, Tokyo, Japan) was inserted followed by selective catheterization of the uterine artery with a high-flow microcatheter. The uterine arteries were embolized with Embospheres (Merit Medical Systems, South Jordan, UT, USA) or coils (COOK Medical Bloomington, Indianapolis, IN, USA) until complete stasis was achieved, defined as a static contrast column.

### 2.4. Definition of Anemia, PPH and RBC Transfusion

In the present study, anemia was defined according to the World Health Organization (WHO) of anemia. Here, anemia is defined as a Hb concentration < 11 g/dL in pregnant women [22]. In Germany, PPH is defined as a blood loss > 500 mL after vaginal delivery and >1000 mL after caesarean section [11]. Transfusion of RBC was in accordance with the German transfusion guidelines. Briefly, RBC transfusion is recommended in asymptomatic patients with Hb < 6 g/dL, in patients with cardiovascular risk factors with Hb between 6 and 8 g/dL or in patients with clinical symptoms of anemic hypoxia [23]. 

### 2.5. Statistical Analysis

Statistical analysis and figure editing were performed using SigmaPlot version 12 (Systat Software, Erkrath, Germany) and GraphPad Prism (Version 7, GraphPad Software Inc., La Jolla, CA, USA). For group comparisons, the one-way ANOVA test (odds ratio; confidence interval 95%) or the unpaired t-test was used. Values were expressed as the number (percentage), mean ± standard deviation (SD), or median (interquartile range (IQR: 25–75)), as appropriate. Uni- and multivariate analyses were performed to evaluate the influence of dependent variables on outcome parameters. All tests were two sided and a *p*-value of <0.05 was considered to be statistically significant.

## 3. Results

Between January 2012 and September 2023, 17 women with PAS underwent primary procedure (caesarean delivery and subsequent UAE) at the University Hospital in Frankfurt. One woman experienced intrauterine fetal death and the placenta was removed during primary procedure. Thus, only 16 women underwent secondary procedure (curettage or HE).

### 3.1. Patient’s Characteristics

The mean age in all women was 35 ±4 years. The median number of gravity was 2.8 (0–7), and the median number of parity was 2.0 (0–5). The median number of prior caesarean sections was 1.3 (0–3). In all women, the preoperative imaging diagnosis of PAS was based on prenatal ultrasound. None of the patients had placenta accreta, 1 (5.9%) woman had placenta increta, and 16 (94.1%) women had placenta percreta. Concomitant placenta previa was present in 11 (64.7%) women. Further characteristics are depicted in Table 1.

### 3.2. Administration of Blood Products, Hb Values and the Anemia Rate

**Figure 1:**
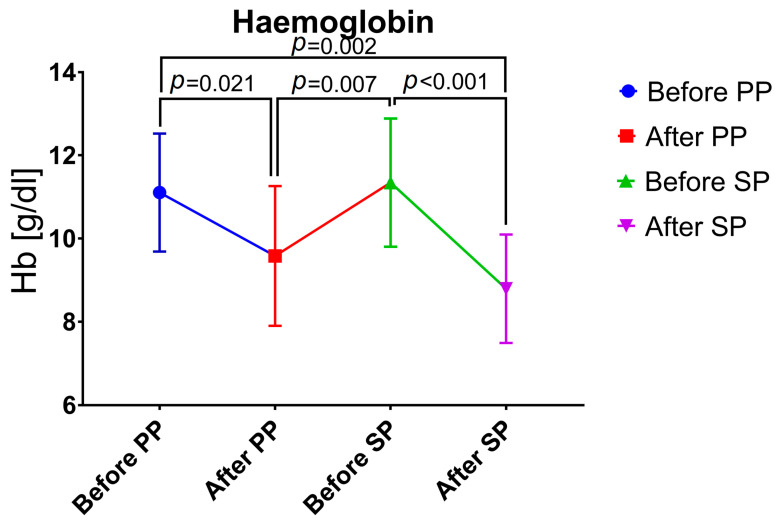
Changes in hemoglobin values. PP, primary procedure; SP, secondary procedure.

At the time of hospital admission for primary procedure, median Hb value was 11.3 (10.9–11.8) g/dL and decreased to 9.7 (8.2–11.1) g/dL at hospital discharge after primary procedure (*p* = 0.021). At the time of hospital admission for secondary procedure, median Hb value increased to 11.5 (10.0–12.4) g/dL (due to RBC transfusions and time to recover between the primary and secondary procedures) and again decreased to 8.4 (7.9–9.5) g/dL at hospital discharge after secondary procedure (*p* < 0.001) (Figure 1). 

Overall, anemia was present in 4 (23.5%) women before primary procedure and 13 (76.5%) women after primary procedure. Anemia rate before secondary procedure was 31.3% (n = 5), after secondary procedure anemia rate increased to 93.8% (n = 15).

Blood products were mostly administered during secondary procedure. In total, 10 (62.7%) women received RBC transfusion during secondary procedure and 13 (76.5%) women received at least one RBC transfusion at any time during hospital stay. Tranexamic acid was administered in 11 (64.7%) women during hospital stay and mostly during the secondary procedure (n= 10; 62.5%). Median blood loss was 300 (200–600) mL during primary procedure and 3600 (450–5500) mL during secondary procedure. Cell salvage was used in three (18.8%) women for curettage or HE (Table 2).

### 3.3. Outcome of Study Population 

**Figure 2:**
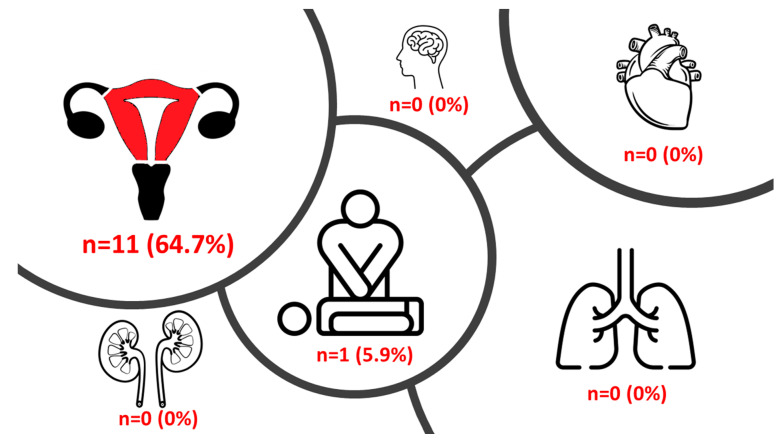
Outcomes in the study population.

Median hospital LOS for the primary procedure was 10.0 (7.5–21.0) days and 6.0 (5.0–10.5) days for the secondary procedure. Only 1 (5.9%) woman suffered from an adverse outcome event (cardiopulmonary resuscitation) during the hospital stay, from which she recovered completely. HE was conducted in 11 (64.7%) women (Figure 2). Univariate analysis revealed blood loss (*p* = 0.025), RBC transfusion (*p* = 0.013), fresh frozen plasma transfusion (*p* = 0.011) and platelet transfusion (*p* = 0.015) to be significantly associated with HE. In multivariate analysis, only the patients’ BMI was significantly associated with anemia and hospital admission for the primary (*p* = 0.020) and secondary (*p* = 0.038) procedures.

Figure 2 depicts the prevalence of the different outcomes in the study population (HE, stroke, cardiac complication/pulmonary embolism, renal failure, resuscitation, and pneumonia).

### 3.4. Anesthesiological and Surgical Management 

For primary procedure, 13 (76.5%) women received epidural anesthesia, 2 (11.8%) women received spinal anesthesia and 2 (11.8%) women received general anesthesia due to contraindication for regional anesthesia. For secondary procedure, in 12 (75.0%) women general anesthesia was conducted. Further anesthesiological procedures are depicted in Table 3. 

Overall, all women (n = 17) received at least one embolization after caesarean delivery. In addition, 3 (17.6%) women received a second embolization due to neovascularization or continuous vaginal bleeding. Embolized vessels during the first embolization included bilateral uterine arteries in 15 (88.2%) women and internal iliac arteries in 2 (11.8%) women.

**Figure 3:**
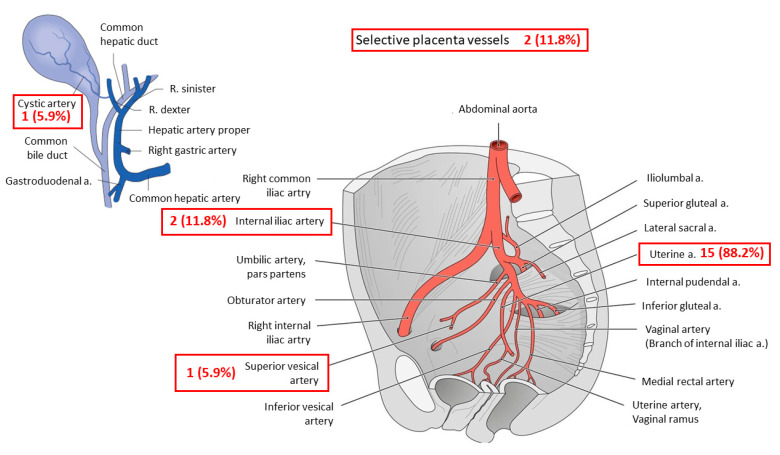
Embolized vessels.

**Figure 4:**
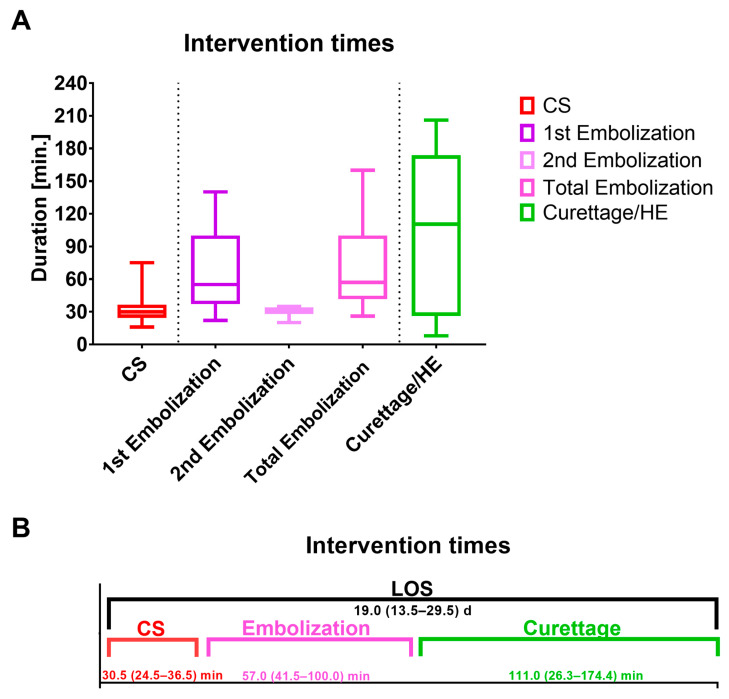
(**A**,**B**) Duration of the primary and secondary procedures.

Further embolized vessels included the cystic artery (n = 1; 5.9%), the superior vesical artery (n = 1; 5.9%) and selective placental vessels (n = 2; 11.8%). In the second embolization, the uterine artery was embolized in 2 (66.7%) women and the internal iliac artery in 1 (33.3%) woman (Figure 3). The mean duration of the first and second embolization was 71.8 (± 39.1) minutes (Figure 4).

Figure 3 depicts the numbers and names of the embolized vessels during the first and second embolization (red boxes). In total, 17 patients underwent one embolization. In 3 patients, a second embolization was necessary. In total, 21 vessels were embolized. A, artery; R, ramus.

Figure 4A depicts the duration of caesarean section and the first embolization (primary procedure) as well as the second embolization and curettage/HE (secondary procedure).

As displayed in Figure 4B, curettage/HE took the longest, with 111.0 (26.3–174.0) min. The median LOS for both procedures together was 19.0 (13.5–29.5) days. CS, caesarean section; HE, hysterectomy; LOS, Length of stay.

## 4. Discussion

This retrospective observational study includes a cohort of 17 women with PAS who underwent a two-step surgical approach: caesarean delivery with subsequent UAE (primary procedure) and placenta removal via curettage or HE (secondary procedure) at the University Hospital in Frankfurt. The main findings of our study are the use of regional anesthesia in 88.2% for caesarean delivery and subsequent UAE and a HE rate of 64.7%. Median blood loss was 300 (200–600) mL for primary procedure and 3600 (450–5500) mL for secondary procedure. The RBC transfusion rate was 11.8% during primary procedure and 62.5% during secondary procedure.

Optimal management of women with PAS involves a standardized approach with a comprehensive multidisciplinary care team. The American College of Obstetricians and Gynecologists (ACOG) guidelines endorse the utility of hysterectomy as standard of care for women with PAS [12]. Controversially, the International Federation of Gynecology and Obstetrics guidelines (FIGO) advocate uterine-preserving surgery whenever possible [24].

Recent studies have reported that pelvic artery catheterization and embolization are safe and effective to prevent HE in women with PAS [25,26]. A study by Munoz et al. in 2023 demonstrates that UAE increases perioperative outcome and reduces maternal morbidity. However, the authors state that with UAE operative times were longer (416 vs. 187 min; *p* < 0.01) and patients were more likely to receive general anesthesia (80 vs. 47%; *p* < 0.01) compared to standard care [27]. In our study, mean times for caesarean delivery (33.5 (±13.7) minutes) and UAE (71.8 (±39.1) minutes) were shorter. Regarding anesthesiological management, only two (11.8%) women undergoing primary procedure required general anesthesia (both due to thrombocytopenia). 

There is evidence that postoperative complications, such as endometritis, urinary voiding difficulties or vascular complications, are more frequent in women undergoing UAE [28]. Complications may lead to uterine necrosis, endometrial atrophy and amenorrhea that are related to wide intrauterine synechia. In addition, infection, leukocytosis and fever (e.g., as part of a postembolization syndrome) may occur [29]. In a study by Alanis et al. including 73 women with PAS endometritis, uterine necrosis and synechia occurred in 8 women (11.0%) following UAE [30]. In our study, none of the above-mentioned complications were observed. The HE rate in the present study was high (64.7%). The aim of UAE is to prevent PPH [11]. Due to the nature of PAS, morphogenic changes and vascularization of the placenta may occur. Despite further embolizations of additional small vascular branches (n = 3 women in our study), secondary procedure may lead to PPH with HE. As only one women suffered from postoperative complications, UAE may still be defined as successful.

A pre- and post-implementation analysis of Munoz et al. of UAE revealed a decreased blood loss by 33% (2000 vs. 3000 mL; *p* = 0.03) and decreased RBC transfusion rate by 51% (odds ratio (OR) 0.05 [95% CI 0.001–0.20]; *p* < 0.01) [27]. In contrast, a comparative study on women with PAS undergoing cesarean delivery with and without subsequent UAE reveals that UAE was associated with a higher blood loss than without UAE (2000 (1500–3000) vs. 1000 (600–2000) mL; *p* = 0.001) [28]. Due to the two-step approach in the present study, median blood loss was only 300 (200–600) mL for caesarean delivery and UAE. However, for secondary procedure, median blood loss was higher (3600 (450–4500)) mL. In addition, RBC transfusion rate was 11.8% during the primary procedure and 62.5% during secondary procedure.

Results of the present study demonstrate that anemia rate prior to delivery was 23.5% and significantly increased by the time of hospital discharge after primary (76.5%; *p* = 0.021) and secondary procedure (93.8%; *p* = 0.002). The findings of an anemia rate prior to delivery of 23.5% are in line with an anemia rate of 23.7% in a recently published study on >6 million pregnant women in Germany [31]. Since data on anemia rates after a two-step approach are scarce, comparison with other studies is not feasible. In a study by Mohr-Sasson et al., the median Hb value after UAE was 7.9 (7.05–9.0) g/dL [28]. These Hb values are comparable to the findings of this study after primary (9.7 (8.2–11.1) g/dL) and secondary procedure (8.4 (7.9–9.5) g/dL).

Antepartum anemia is common and mainly caused by increased iron demands during pregnancy [32]. However, postpartum anemia may also occur in 50–80% of all women. Iron deficiency and increased peripartum blood losses are the main causes of postpartum anemia and a potentially preventable condition [33]. National and international guidelines strongly recommend the diagnosis of antepartum and postpartum anemia [34,35]. Screening for hematological conditions with a full blood count should be conducted at 28 weeks of gestation, as well as at any time during pregnancy if anemia is present [34,35]. Given the fact that antepartum iron deficiency is a modifiable risk factor for anemia in the postpartum period, routine antenatal administration of oral iron (30–60 mg/day) and folic acid (400 μg/day) is recommended. Mild postpartum anemia should be treated with oral iron (Hb 9.0–11.0 g/dL); in moderate to severe postpartum anemia (Hb < 9 g/dL), treatment with intravenous iron is recommended [34]. To reduce intraoperative blood loss and peripartum transfusion rates, international guidelines strongly recommend the use of cell salvage [36]. The use of cell salvage should be especially considered in women with anticipated high risk for severe hemorrhage (e.g., PAS) or in case of unanticipated bleeding during surgery [37], along with other measures such as prophylactic use of tranexamic acid [38]. A recent study on the use of cell salvage in women undergoing caesarean section with PPH in Germany revealed that cell salvage is used in only 0.07% of all bleeding women. Thus, there is tremendous potential to increase the use of blood conservation strategies [39].

Last, for primary and secondary procedure general anesthesia was performed in 11.8%and 75.0% of the women, respectively. PPH is often caused by uterine atony. Volatile agents cause dose-related relaxation of the uterus, which may lead to increased blood losses. After delivery, volatile anesthetics should be decreased and oxytocin should be given to decrease uterine relaxation and blood loss [40]. Therefore, it is noteworthy to mention that the anesthesiologic procedure and use of agents may also have an impact on the magnitude of blood loss during both procedures.

## 5. Limitations

The present study has some limitations. First of all, due to the retrospective nature of this study, allocation of women diagnosed with PAS and treatment were not randomized. Therefore, results might have been biased by patient treatment selection. In addition, the present study included women over a period of 12 years due to the rarity of PAS and the need to achieve a proper sample size. However, an increased sample size of women with PAS undergoing UAE could lead to more robust statistical analyses. During the observation time, advances in surgical and anesthesiological management were substantial, aiming for the embolization of smaller branches of the uterine artery and the implementation of blood conservation strategies. In addition, since these procedures have been conducted at our institution for >10 years, there is no control group for treatment of women diagnosed with PAS and different treatment strategies other than UAE during the primary procedure and placenta removal for the secondary procedure. In addition, as this is a single-center experience with an established treatment algorithm of women with PAS, comparison of results may be difficult. Last, to increase study quality and the number of included women with PAS, a multicenter approach should be considered in future studies. In summary, the present study evaluated a small patient population undergoing a highly specialized and standardized procedure. The surgical two-step approach with delivery, subsequent UAE and curettage/HE is very unique and an established procedure over the years at our institution. Despite the small sample size and the above-mentioned limitations, this report may serve as an example of treatment of women with PAS for hospitals worldwide.

## 6. Conclusions

The analysis of 17 women undergoing a two-step approach for treatment of PAS revealed that for caesarean delivery and subsequent UAE, the use of regional anesthesia (spinal or epidural anesthesia) is safe. After primary (76.5%) and secondary procedure (93.8%), postpartum anemia rate was high. In total, 62.7% of women received RBC transfusion during secondary procedure. Anemia management and the implementation of blood conservation strategies are crucial in women diagnosed with PAS undergoing UAE.

## Figures and Tables

**Table 1:** Characteristics of the study population.

Characteristic n (%)	
Age (years)	35 ± 4 ^#^
BMI (kg/m^2^)	28 ± 7 ^#^
ASA score	II: 12 (70.6%) III: 5 (29.4%)
Gravity	2.8 (0–7) *
Parity	2.0 (0–5) *
Prior caesarean deliveries	1.3 (0–3) *
Singelton	17 (100%)
**Placenta n (%)**	
Bladder invasion	9 (52.9%)
Prenatal imaging diagnosis Placenta accreta Placenta increta Placenta percreta	0 (0%) 1 (5.9%) 16 (94.1%)
Concomitant placenta previa	11 (64.7%)
**Comorbidities n (%)**	
Pre-eclampsia/Eclampsia	0 (0%)
HELLP	0 (0%)
Gestational diabetes	3 (17.6%)
Gestational hypertension	0 (0%)
Thrombocytopenia	2 (11.7%)

BMI, body mass index; ASA, American Society of Anaesthesiologists; HELLP, hemolysis, elevated liver enzymes, and low platelet count. ^#^ Results presented as the mean (±SD). * Results presented as the median (IQR: 25–75).

**Table 2:** Utilization of blood products and transfusion rates.

	A. Primary Procedure (n = 17)	B. Secondary Procedure (n = 16)	C. Ward (n = 17)	D. Total (n = 17)	*p*-Value
RBC Units *	0.0 (0.0–0.0)	2.0 (0.0–5.5)	0.0 (0.0–3.0)	4.0 (0.5–7.0)	A. vs. B.: *p* = 0.038
Transfusion rate n (%)	2 (11.8%)	10 (62.5%)	8 (47.1%)	13 (76.5%)	A. vs. D.: *p* = 0.002
FFP Units *	0.0 (0.0–0.0)	0.0 (0.0–4.0)	0.0 (0.0–0.0)	0.0 (0.0–4.5)	
Transfusion rate n (%)	0 (0%)	6 (37.5%)	1 (5.9%)	6 (35.3%)	
Platelets Units *	0 (0–0)	0 (0–1.0)	0 (0–0)	0 (0–2.5)	
Transfusion rate n (%)	0 (0%)	5 (31.3%)	2 (11.8%)	7 (41.2%)	
Fibrinogen (2 g) *	0.0 (0–0)	1.0 (0–4.0)	0 (0–0)	2 (0–4.0)	A. vs. B.: *p* = 0.024
Administration n (%)	1 (5.9%)	8 (50.0%)	3 (17.6%)	10 (58.8%)	A. vs. D.: *p* = 0.003
PCC (Units) *	0 (0–0)	0 (0–0)	0 (0–0)	0 (0–0)	
Administration n (%)	0 (0%)	3 (18.8%)	0 (0%)	3 (17.6%)	
TXA [mg] *	0 (0–500.0)	1000 (0–2000.0)	n.a.	1000 (0–2000)	A. vs. D.: *p* = 0.010
Administration n (%)	4 (23.5%)	10 (62.5%)	n.a.	11 (64.7%)	
Cell Salvage n(%)	0 (0%)	3 (18.8%)	0 (0%)	3 (18.8%)	
Blood Loss (mL) *	300 (200–600)	3600 (450–5500)	n.a.	1500 (600–4500)	A. vs. B.: *p* = 0.008 A. vs. D.: *p* = 0.006

RBC, red blood cell; FFP, fresh frozen plasma; PCC, prothrombin complex concentrate; TXA, tranexamic acid; n.a., not available. * = Results displayed as the median (IQR: 25–75).

**Table 3:** Anesthesiological management for the primary and secondary procedures.

Anesthesiological Procedure	Primary Procedure (n = 17)	Secondary Procedure (n = 16)
Epidural	13 (76.5%)	4 (25.0%)
Spinal	2 (11.8%)	0 (0%)
General anesthesia	2 (11.8%)	12 (75.0%)
Arterial line	10 (58.8%)	10 (62.5%)
Central venous line	2 (11.8%)	9 (56.3%)

## Data Availability

The data cannot be shared publicly due to national data protection laws.
